# Degradation Behavior of Polypropylene during Reprocessing and Its Biocomposites: Thermal and Oxidative Degradation Kinetics

**DOI:** 10.3390/polym12081627

**Published:** 2020-07-22

**Authors:** Elnaz Esmizadeh, Costas Tzoganakis, Tizazu H. Mekonnen

**Affiliations:** Department of Chemical Engineering, Institute of Polymer Research, University of Waterloo, Waterloo, ON N2L 3G1, Canada; elnaz.esmizadeh@uwaterloo.ca (E.E.); costas.tzoganakis@uwaterloo.ca (C.T.)

**Keywords:** Polypropylene, recycling, degradation kinetics, thermogravimetric analysis, wood–plastic composites

## Abstract

Non-isothermal thermogravimetric analysis (TGA) was employed to investigate the degradation of polypropylene (PP) during simulated product manufacturing in a secondary process and wood–plastic composites. Multiple batch mixing cycles were carried out to mimic the actual recycling. Kissinger–Akahira–Sunose (KAS), Ozawa–Flynn–Wall (OFW), Friedman, Kissinger and Augis models were employed to calculate the apparent activation energy (*E_a_*). Experimental investigation using TGA indicated that the thermograms of PP recyclates shifted to lower temperatures, revealing the presence of an accelerated degradation process induced by the formation of radicals during chain scission. Reprocessing for five cycles led to roughly a 35% reduction in ultimate mixing torque, and a more than 400% increase in the melt flow rate of PP. *E_a_* increased with the extent of degradation (α), and the dependency intensified with the reprocessing cycles. In biocomposites, despite the detectable degradation steps of wood and PP in thermal degradation, a partial coincidence of degradation was observed under air. Deconvolution was employed to separate the overlapped cellulose and PP peaks. Under nitrogen, OFW estimations for the deconvoluted PP exposed an upward shift of *E_a_* at the whole range of α due to the high thermal absorbance of the wood chars. Under air, the *E_a_* of deconvoluted PP showed an irregular rise in the initial steps, which could be related to the high volume of evolved volatiles from the wood reducing the oxygen diffusion.

## 1. Introduction

Green awareness influences today’s industry significantly in several ways, in order to increase human preference for more eco-friendly procedures and products. Recently, the practice of recycling commodity polymeric materials such as polypropylene (PP) has been encouraged by the ever-growing desire to save resources, reduce cost, and reuse waste material [1]. Among various recycling techniques, the most interesting for economic and environmental reasons is primary recycling, which involves mechanical crushing and reprocessing to obtain new products [2,3]. The effect of PP reprocessing via extrusion [3,4,5,6] and injection molding [7], under intensive shear and elevated temperatures, has been well-documented in the literature. However, the changes in the chemical structure of PP as a result of re-extrusion and re-injection are very limited, as reported by Sarrionandia et al. [8] and Aurrekoetxea et al. [7], revealing zero or limited oxidation as a result of these processes. Concerning thermal stability, Camacho et al. [9] found a continuous decrease in the degradation temperature of PP with reprocessing cycles, while Jimenez et al. [10] indicated no significant differences between virgin and recycled PP in terms of degradation behavior.

Wood–plastic composites (WPC), which typically contain wood (fiber, flour, pulp), plastic and additives (e.g., coupling agents, processing aids), are used to substitute plastics, solid wood and steel in applications such as construction, and in industrial and agricultural products [11,12]. This is mainly because of their low-cost, improved performance and environmental-friendliness attributes [13,14]. Plant biomass reinforcements, such as wood fibers, are biodegradable, inexpensive, renewable, light-weighted, and have good mechanical properties comparable to their alternative (glass fibers) [15,16]. These characteristics make WPCs appealing materials for non-structural constructions, such as decking, fencing, sidings, railings and door profiles [17]. Various grades of polyethylene [13], polypropylene [15,18], polystyrene [19], polyvinyl chloride [20] and poly methyl methacrylate [21] polymers are extensively utilized as WPC matrices.

WPCs can be produced in a variety of shapes via a wide range of thermoplastic manufacturing methods, including extrusion, injection and compression molding [22]. Moreover, WPCs have the ability to thermally decompose more easily, with induced degradability, due to the inherent low thermal stability of wood-based reinforcements, which is desired for reducing the environmental disadvantage [23]. However, the degradation of wood components during the thermo-mechanical processing of WPCs may lead to several undesirable properties in the fabricated products, such as deterioration of color, unpleasant odor, and poor mechanical properties [18,24]. On the other hand, physical and chemical changes on the surface of the bio-based material during degradation can also lead to improved interfacial adhesion between wood and polymer, thereby resulting in superior overall performance. Therefore, an understanding of the degradations of wood and polymer in the presence of each other is an important scientific and engineering challenge that needs to be addressed for WPC production process development. Such understanding will also allow us to effectively utilize recycled commodity polymers, such as PP in WPCs. Many researchers have reported a higher thermal stability in WPCs than in pure wood [25,26]. Heat treating [27] and the extraction of hemicellulose [28] from wood flour were proposed to further improve the thermal stability. Jeske et al. [29] developed a special thermogravimetric analysis (TGA) method, based on the step separation of curves for quantitative analysis in the WPC’s thermal degradation.

To the best of our knowledge, no studies on the effects of the repetitive recycling process, and the presence of plant biomass reinforcement, on the degradation kinetics of PP have yet been reported in the scientific literature. Thus, the objective of this work was to investigate the thermal and oxidative degradation kinetics, using the various models proposed by Kissinger, Kissinger–Akahira–Sunose (KAS), Flynn–Wall–Ozawa (FWO), Friedman and Augis. An internal batch mixer was employed to simulate recycling so to avoid further oxidation. The results of this work have led to the following contributions in the field: (i) elucidating the effect of PP reprocessing and (ii) the effect of plant biomass in PP-based WPC, and (iii) pointing out the importance of the presence of oxygen, providing practical, important information on degradation behavior under more realistic atmospheric conditions.

## 2. Materials and Methods

### 2.1. Materials

Polypropylene homopolymer powder (Pro-fax 6301, melt flow rate (MFR) of 10–12 g/10 min, ASTM D1238) was obtained from LyondellBasell (Houston, TX, USA). Maple wood flour, mesh 600, was supplied by Ontario Sawdust Supplies (East Gwillimbury, ON, Canada) and dried overnight at 100 °C prior to compounding to eliminate moisture. No additives were used in the process of WPC to avoid interference in the degradation kinetics study.

### 2.2. Experimental Methods

The compositions of the wood–plastic composites, shown in Table 1, were dry mixed for several minutes and subsequently compounded in a HAAKE Rheomix 3000 batch mixer (Thermo Fisher Scientific Inc., Waltham, MA, USA) at the mixing temperature of 180 °C and rotor speed of 80 rpm. Once a constant torque value was obtained, the sample was cooled to room temperature. Compounds were ground into small particles using a laboratory mill to obtain uniform ground segments. In-situ rheological properties were recorded from the evolution of torque with time during processing. The effects of reprocessing on the melt viscosity, and indirectly on the molecular weight, were analyzed by MFR measurements, using a Kayeness Galaxy V capillary rheometer apparatus (Morgantown, PA, USA), according to ASTM D1238-68T at 230 °C and 2.16 kg. Measurements were also repeated at a temperature of 190 °C to evaluate the effect of temperature, since the flow rate was too high for multiple-reprocessed PP. Thermogravimetric analyses were performed on a TA instrument Q500 analyzer (New castle, DE, USA) under both nitrogen and air atmospheres, with a 40 mL/min flow. Dynamic non-isothermal experiments were conducted using four heating rates of 5, 10, 20 and 40 °C/min from ambient temperature to 700 °C. The weight of samples was kept about 15 ± 2 mg.

### 2.3. Theoretical Background

For a non-isothermal TGA run, the fractional extent of decomposition conversion (α) at any temperature (*T*) is directly proportional to weight loss, and can be calculated using Equation (1):

(1) $$\alpha =\frac{m_{i}-m}{m_{i}-m_{f}}=1-wt_{loss}$$

where *m*_i_ is the initial mass, m is the momentary mass at each instant time and temperature, *m*_f_ is the final mass and *wt_loss_* is the weight loss at any temperature [2 3]. The conversion rate, following the kinetics, in the solid-state can be described as the first derivative of conversion by Equation (2):

(2) dαdx=k(T)f(α)=Ze−EaRT f(α)

where k(T) is the kinetic constant generally expressed by the Arrhenius equation. *Z* is the pre-exponential factor, $E_{a}$ is the apparent activation energy, *T* is the absolute temperature and *R* is the gas constant. f(α) is the governing reaction model, which is a function of the reaction mechanism pronouncing the physical or chemical changes during the degradation reaction.

The value of the activation energy ($E_{a}$), which is essential in evaluating the most suitable kinetic model for the degradation process, can be determined from the slope of a plot of two parameters given by the mathematical equations of the models. Several methods [30,31], which were employed to calculate the $E_{a}$ values at progressive degrees of conversion, are given in Table 2. Linear regression was performed to determine the $E_{a}$ from the slope of curves (mentioned in the table) based on taking the adjusted *R*-square value as the criterion in order to get the descriptive power of regression.

## 3. Results and Discussion

### 3.1. Effect of Reprocessing Cycles

**Figure 1:**
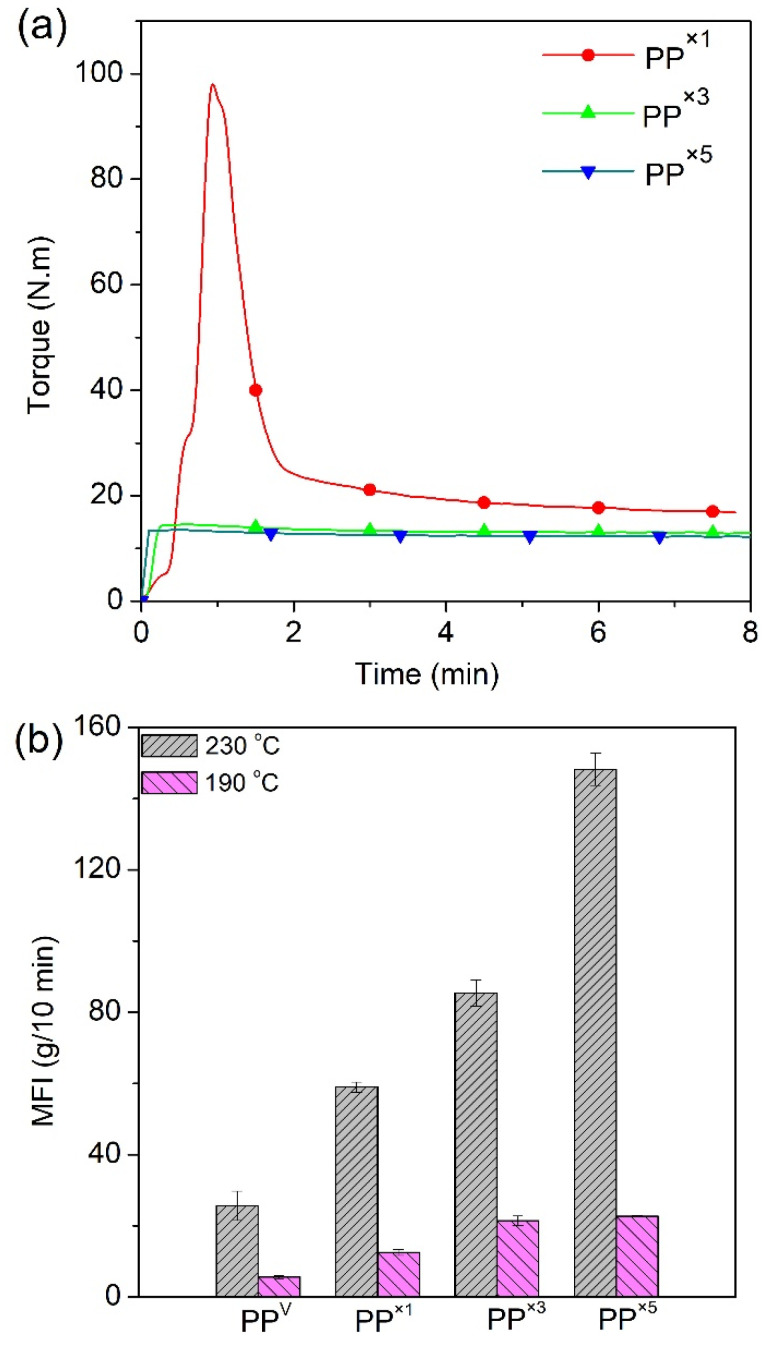
Processing of polypropylene. (**a**) Evolution of torque and (**b**) MFR of PP as a function of the number of processing cycles.

In order to study the effect of the multiple processing of PP on its viscosity, the evolution of torque during processing in the batch mixer was recorded, and the results are shown in Figure 1a for different reprocessing cycles. In the first PP processing cycle, a typical major melting peak was observed right after material loading up until the polymer reached its melting point, due to shearing between the rotors and the inner wall of the mixing chamber [32]. Subsequently, due to the shear-thinning properties of PP, the torque started to drop gradually until the system achieved a steady-state and the mixing process was considered as completed. A distinguishable decrease in the ultimate torque (*τ_∞_*) or steady-state torque value was observed as the number of reprocessing cycles increased (Figure 1a). *τ_∞_* decreased with the reprocessing cycle, from 17 N∙m for one-time processed PP (PP^×1^) to 13 and 11 N∙m for PP^×3^ and PP^×5^, respectively. *τ_∞_* is an indication of the viscosity of the melt at a given temperature, reflecting the molecular weight variation with reprocessing cycles. The reduction in *τ_∞_* is predominately due to molecular weight reduction induced by chain scission, resulting from the mechanical shearing action in the process.

No significant chemical structure changes, or formations of peaks associated with the oxidation of PP after several reprocessing cycles, were noted here (Supporting Information Figure S1). This has been extensively investigated and reported in the literature using Fourier-transform infrared spectroscopy (FTIR) and other analytical techniques [7,33]. This behavior is explained by the absence of oxygen within the polymer matrix causing oxidative degradation during reprocessing, suggesting that other chain scission, and not oxidation, is the dominant degradation mechanism for the recycled PP. As expected, the higher the number of cycles, the lower the ultimate torque value and the molecular weight [4]. Noticeably, the main reduction in *τ_∞_* occurred after the first reprocessing cycle, and with further processing, *τ_∞_* becomes less sensitive to the reprocessing cycle. The major cause of such observation is the well-known fact that the higher the molecular weight, the greater the mechanical degradation during the process [32]. Thus, the breakdown of the molecular chain in the first few cycles is predominately controlled by scission induced by the mechanical shear force. After several reprocessing cycles, the mechanical decomposition becomes less important, and thermal decomposition may dominate. This observation reflects the milder decomposition effect of thermal decomposition, compared to the mechanical decomposition. The molecular weight drop during the reprocessing of PP can be also accounted for by an increase in the melt flow index with the reprocessing cycles. Figure 1b shows that the values of MFR for PP increased as a function of the number of reprocessing cycles, indicative of the molecular weight loss of the polymer matrix during reprocessing [34]. Research findings have shown an inverse relation between MFR and apparent viscosity, which directly relates to chain length [35]. Between zero and five cycles of reprocessing, the MFR value increased by 470% and 300%, at 230 °C and 190 °C, respectively, indicating that at higher MFR test temperatures, repetitive reprocessing has a higher influence on MFR. Such a tendency of increase in MFR at higher temperatures is again indicative of the importance of thermo-degradation. The *τ_∞_* and MFR of some other recyclates (PP^×2^ and PP^×6^) are presented in line with the other results (Supporting Information Figure S2).

**Figure 2:**
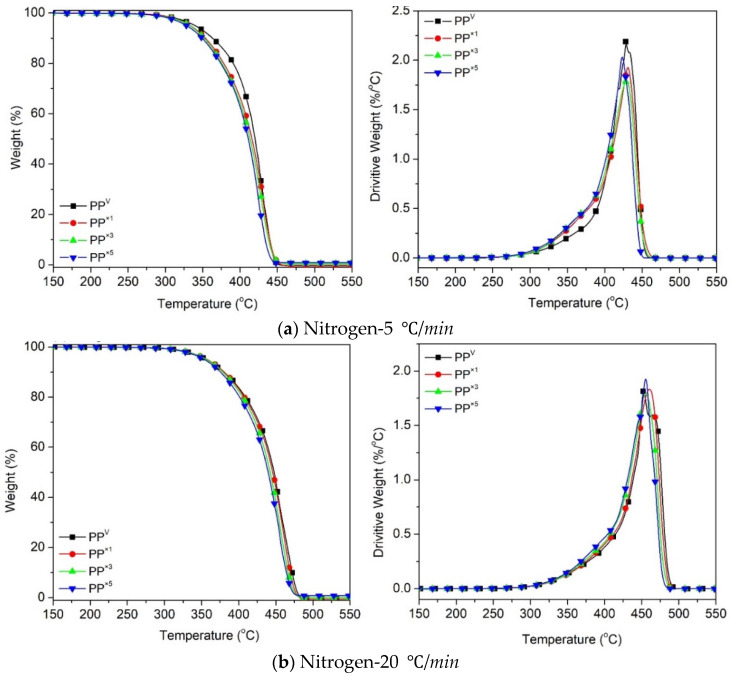
Thermogravimetric (TG) and first-order derivative (DTG) curves for the effect of reprocessing cycles on the thermal decomposition of PP at heating rates of: (**a**) 5 °C/min, (**b**) 20 °C/min.

**Figure 3:**
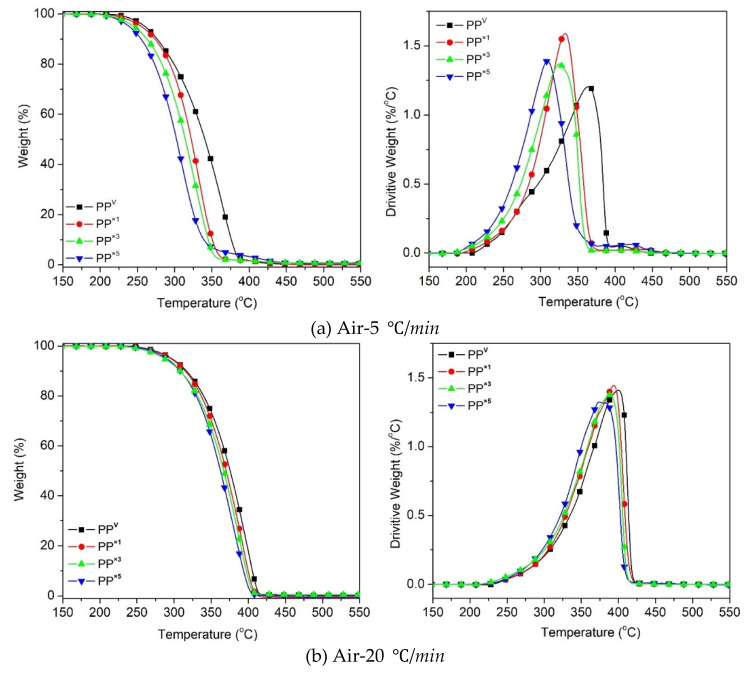
TG and DTG curves for the effect of reprocessing cycles on the oxidative decomposition of PP at the heating rates of: (**a**) 5 °C/min, (**b**) 20 °C/min.

**Figure 4:**
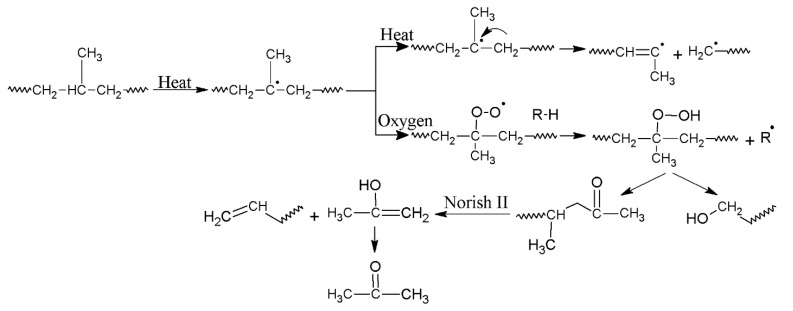
Schematic illustration of the various reactions that may occur during thermal and oxidative degradation of PP [36].

Figure 2 shows the thermogravimetric (TG) and the first-order derivative (DTG) curves, at a heating rate of *β* = 5 °C/min and 20 °C/min, of the virgin PP and the samples reprocessed via batch mixing under a non-oxidative environment. Nevertheless, the presence of oxygen as a degrading agent during any step of the material loop (processing, service life, discarding and recovery) can influence the thermal degradation stability and mechanisms. Figure 3 displays the TG/DTG data of PP and its recyclates degraded in the presence of oxygen. The most commonly proposed mechanism for the thermal and oxidative degradation of PP is illustrated in Figure 4 [36]. Similar curves were obtained for the samples at other heating rates (Supporting Information Figure S3). As expected, increasing the heating rate led to a shift of the thermograms towards higher temperatures, both in oxidative and non-oxidative degradation. This is due to the shorter retention time at the higher heating rates, which limits the time-consuming molecular motions taking place before decomposition [37]. In other words, the degradation reaction is influenced by the factors of time and temperature simultaneously. As can be observed in Figure 2 and Figure 3, all curves exhibit one-step degradation that can be attributed to the radical random scission commonly occurring with the thermal degradation of the polyolefins [3]. In a nitrogen environment, PP degrades in a single step, beginning at 300 °C and ending at 475 °C. On the other hand, it degrades from about 250 to 425 °C, primarily in a single step, in the presence of air. Under both environments, all PP samples degraded completely, without leaving any significant residue. It can also be clearly observed that the thermo-oxidative decomposition took place at lower temperatures, compared with the inert environment. Besides, the degradative effect of the reprocessing cycles is more pronounced in the experiments in the air. This can be attributed to the formation of unstable proxy radicals and hydroperoxides (see Figure 4) with oxygen uptake, which at high temperatures rapidly convert into other labile products as they evolve volatile compounds [36].

To assess the thermal stability of PP and its reprocessed recyclates, the corresponding decomposition onset temperature (*T_onset_*) and the temperature at the maximum decomposition rate, i.e., the peak temperature (*T_peak_* or *T_max_*), obtained from the TG and DTG curves, were tabulated (Table 3). As the identification of *T_onset_* at conversions near zero was difficult, the temperature related to 5% weight loss was considered *T_5%_*, in order to provide an overview of the thermal stability. It can be observed that in all the PP systems, increasing the heating rate increased *T_5%_* and *T_peak_*. It was also noted that the characteristic temperatures, *T_5%_* and *T_peak_*, obtained for reprocessed PP were lower than those of the virgin PP, suggesting that reprocessed PP samples were quicker in reaching the exothermic peak than neat PP. The scission of the PP chains, and the formation of radicals during reprocessing induced by thermo-mechanical degradation, is the reason for the appearance of new sites liable to decomposition, which promotes an accelerated degradation process at high reprocessing cycles. The shorter the chains, the more prone they are to thermal and oxidative degradation at lower temperatures [36]. The obtained result was in agreement with the abovementioned higher MFR, as a consequence of the chain scission mechanism.

**Figure 5:**
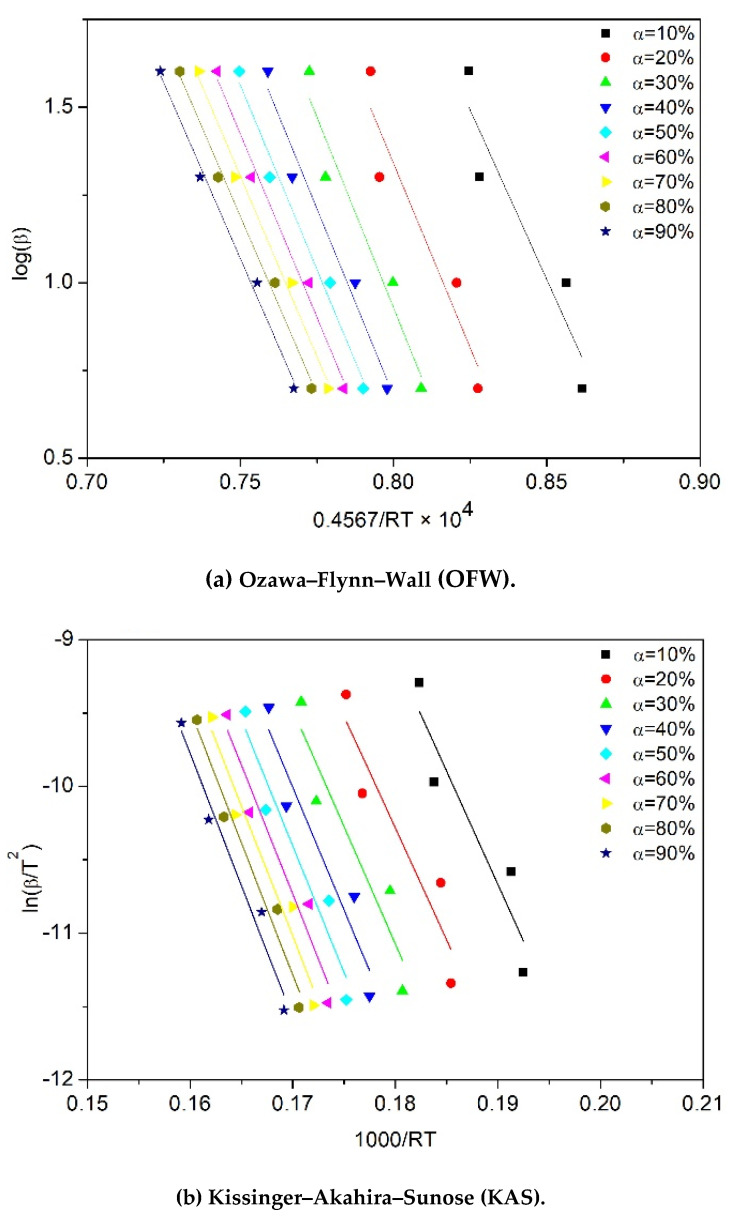

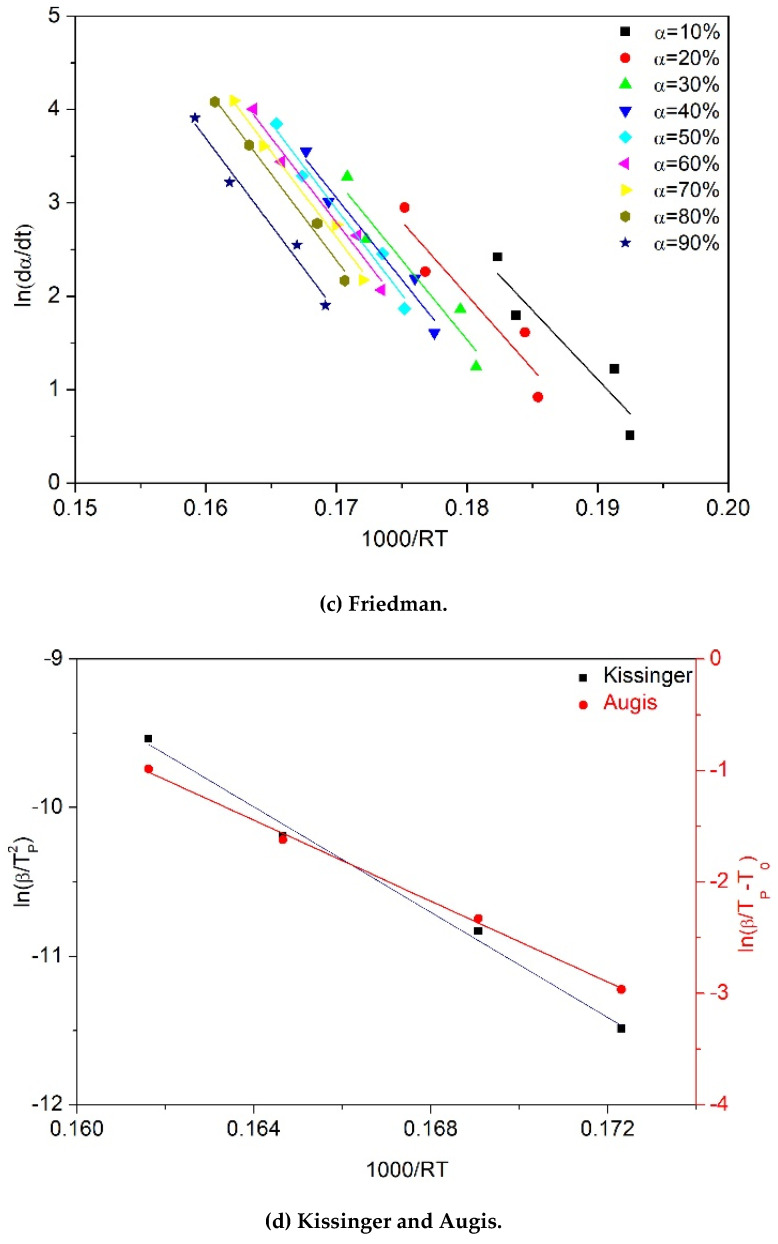
Plots required to calculate activation energy (*E_a_*) for PP^×1^ sample according to different kinetic equations: (**a**) OFW, (**b**) KAS, (**c**) Friedman, (**d**) Kissinger and Augis.

To calculate the activation energy ($E_{a}$) of the degradation process by using the equations shown in Table 2, the following curves were plotted for each sample: $\ln\left(\frac{\beta }{T^{2}}\right)$ versus $\frac{1}{RT}$ for the KAS model, $\ln\left(\frac{\beta }{T_{P}{}^{2}}\right)$ versus $\frac{1}{RT_{P}}$ for the Kissinger model, logβ versus $\frac{0.4567}{RT}$ for the OFW model, $\ln\left(\beta \frac{d\alpha }{dT}\right)$ versus $\frac{1}{RT}$ for the Friedman model, and $\ln\left(\frac{\beta }{T_{P}-T_{0}}\right)$ versus $\frac{1}{RT_{P}}$ for the Augis model. For instance, the curves plotted for the thermal degradation of one-time processed PP (sample PP^×1^) are presented in Figure 5. The activation energy values were found by linear regression of the plotted data. The regression coefficient was mostly above 90% confidence for all PP samples. Noticeably, the variation in the slopes of the fitted lines (Figure 5a–c) illustrates the dependency of the activation energy on the conversion in OFW, KAS and Friedman models. In contrast, one unique activation energy can be calculated from the conversion-independent models, Kissinger and Augis (Figure 5d).

**Figure 6:**
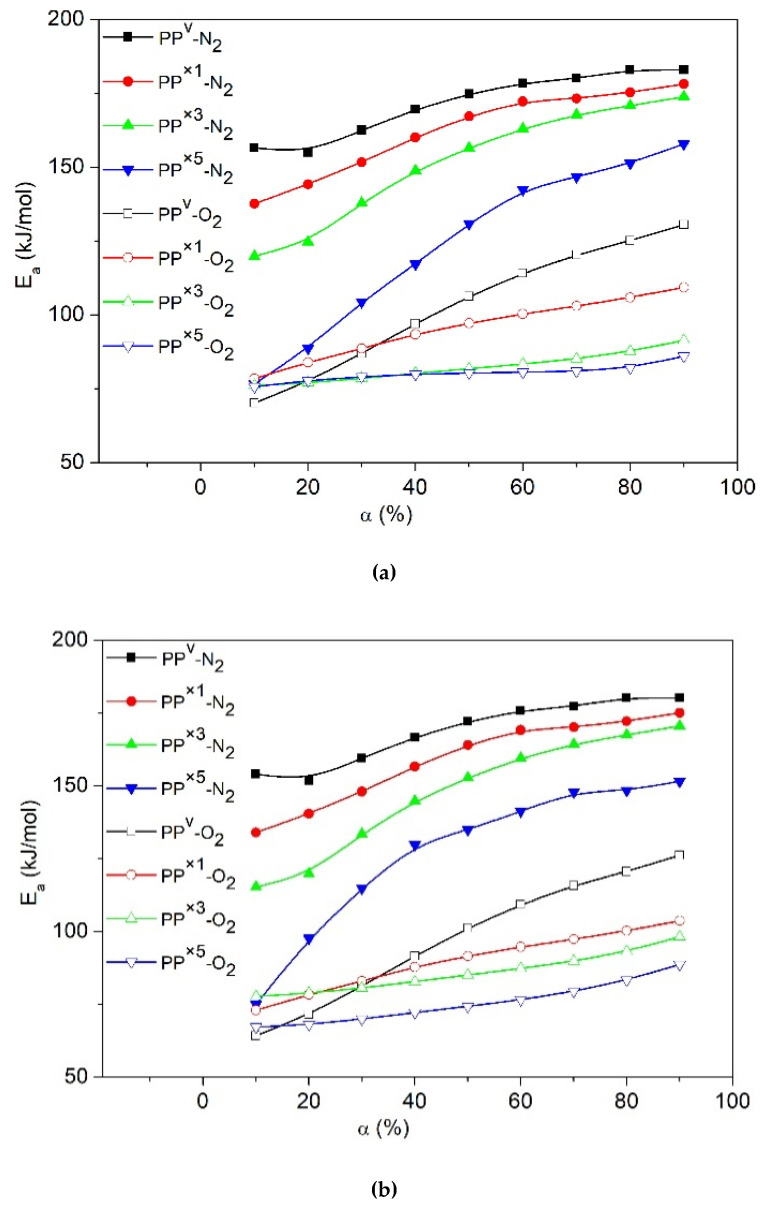

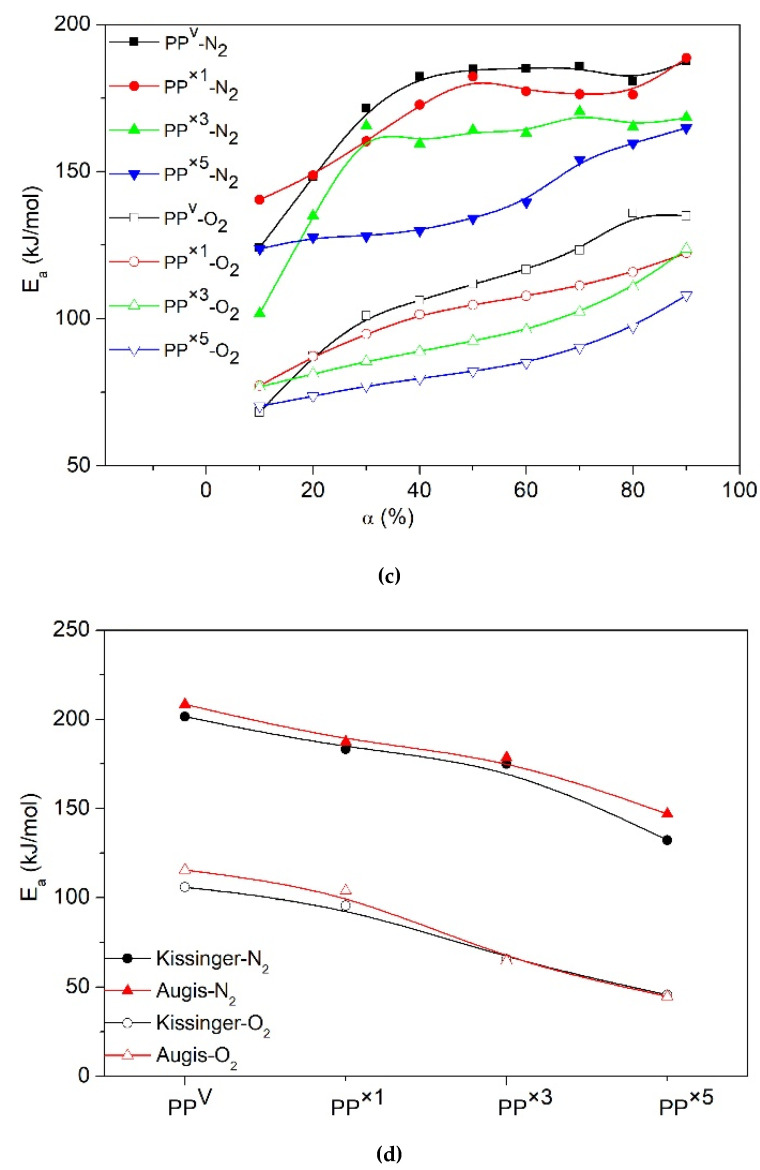
Evolution of activation energies (*E_a_*) as a function of the degree of conversion (α) for virgin PP and its recyclates obtained by different models (**a**) KAS, (**b**) OFW, (**c**) Friedman, (**d**) Effect of the number of reprocessing cycles on *E_a_* according to Augis/Kissinger models.

The slopes of the fitted curves were calculated, and the evolution of *E_a_* values versus α for virgin PP and its recyclates decomposed under N_2_ and the air were demonstrated, in Figure 6a–c. A gradual increase in the *E_a_* values with an increase in the degree of conversion was observed for all the samples. For instance, according to OFW, the *E_a_* of virgin PP increases from ~150 kJ∙mol^−1^ at low conversions to ~190 kJ∙mol^−1^ at the end of the decomposition. Such a large deviation in the *E_a_* values suggests consideration of the variable *E_a_* for calculation of the pre-exponential factor (*Z*) and the governing reaction model (*f*(α)) in kinetics, instead of applying simple kinetic models with constant activation parameters. Lower *E_a_* values at early stages of degradation suggest that at low conversions decompositions, kinetics were limited by initiation at the weak links. The increasing trend with the evolution of *E_a_* can be interpreted via a shift in the rate-limiting step, from initiation at the weak links to the degradation initiated by random scission [36]. Vyazovkin et al. explained the increase of *E_a_* throughout the conversion via the fact that the decomposition of the residual material consistently becomes more stable as the temperature rises [38].

Under the air, the activation energy of PP is about ~70 kJ∙mol^−1^ (Figure 6a–c) during the early stages of degradation, which agrees well with the activation energy range reported for the decomposition of peroxides [39]. This observation suggests that the degradation kinetics at low conversions are limited by the peroxide radical decomposition. For the later degradation stages, the activation energy increases to values around ~160 kJ∙mol^−1^ (Figure 6a–c), like the trend observed for thermal degradation at high conversions. It appears that oxidative degradation of PP proceeds with a change in the rate-limiting step, from peroxy radical decomposition to the degradation initiated by random scission [36].

These results also allow us to conclude that reprocessed recyclates with higher MFR had lower activation energies when compared to the virgin PP. Concerning the evolution of *E_a_*, a progressive reduction was shown along with the successive reprocessing cycles. Once PP is exposed to repetitive reprocessing, the long backbones of the molecular chain break continuously, resulting in progressively lower molecular weight species with some new sites vulnerable to decomposition. Low molecular weight chains need less energy for scission, and can reach the evaporation temperature without degradation or by several chain scissions [40]. This evaporation phenomenon could act as a process aid, making PP chains move and vibrate more easily in the polymer bulk, thereby decreasing the viscosity (reflected in high MFR values and low *τ_∞_* above). Such movements increase the probability of random contacts in the polymer bulk, and facilitate the cracking of the chain and consequently decrease the activation energy for decomposition. The postulated easier cracking agrees well with our experimental observation that the PP recyclates were degraded at lower temperatures.

### 3.2. Effect of Wood

**Figure 7:**
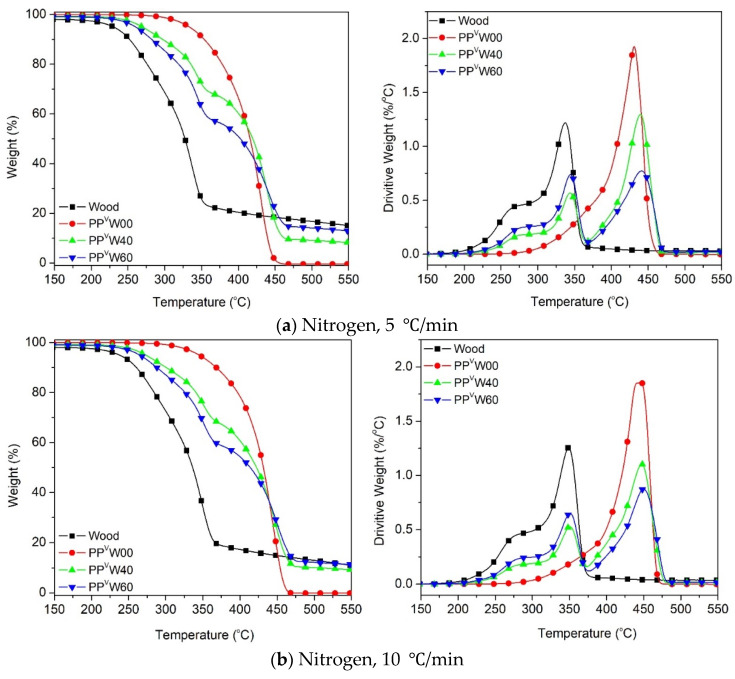

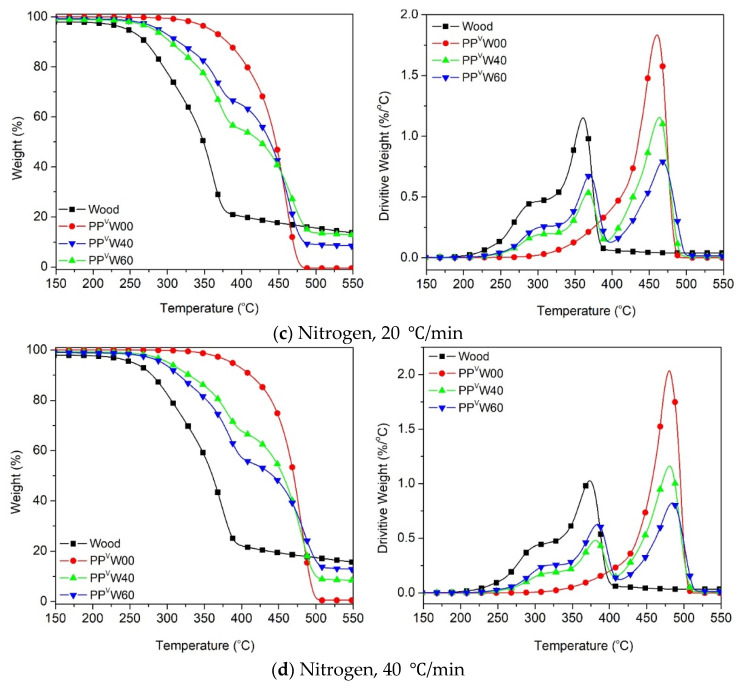
Thermal degradation of wood and PP-based WPCs at different heating rates: (**a**) 5 °C/min, (**b**) 10 °C/min, (**c**) 20 °C/min and (**d**) 40 °C/min.

**Figure 8:**
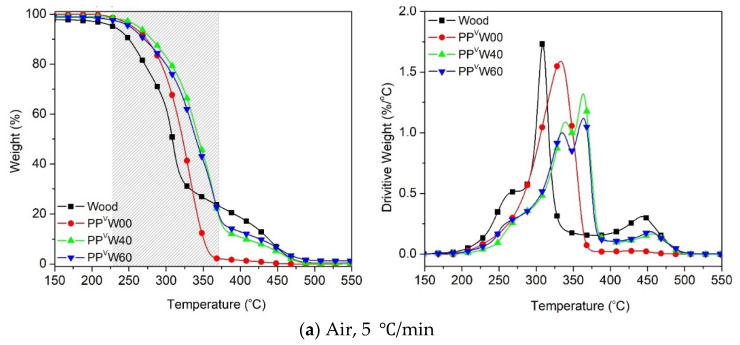

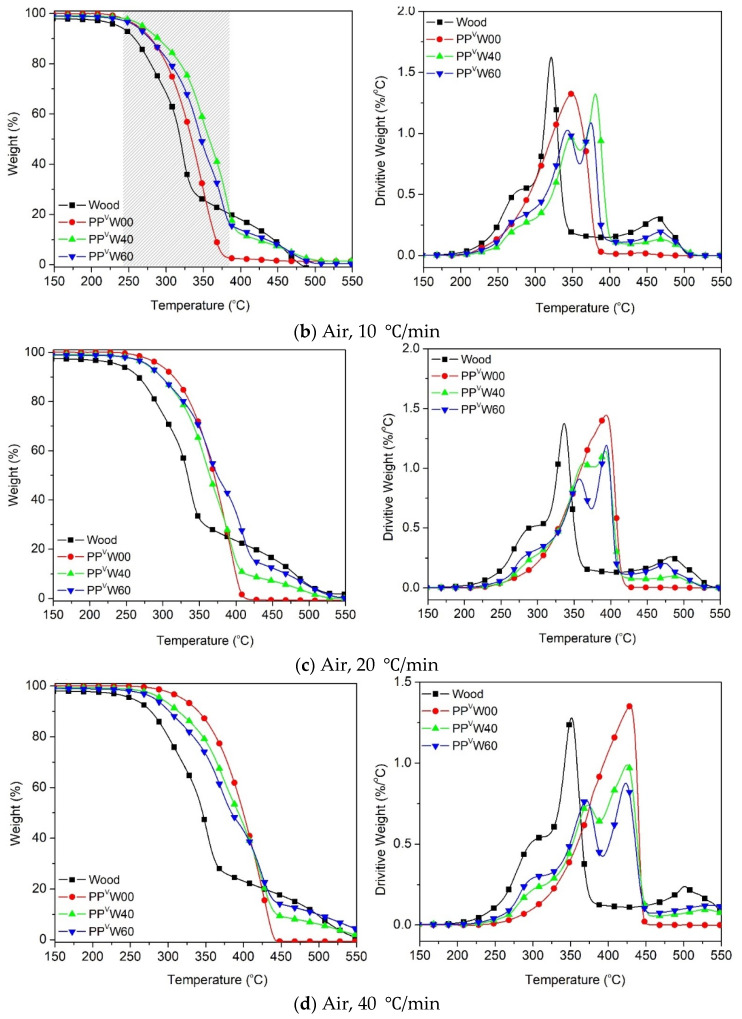
Oxidative degradation of wood fiber and PP-based WPCs at different heating rates: (**a**) 5 °C/min, (**b**) 10 °C/min, (**c**) 20 °C/min and (**d**) 40 °C/min.

To evaluate the degradation behavior of a PP-based WPC, TGA evaluations were conducted. The degradation characteristics, both TGA and DTG curves of the wood fiber and PP-based WPCs in nitrogen and air atmospheres, are presented in Figure 7 and Figure 8, respectively. It can be observed that the decomposition temperature for each sample varies depending on the experimental conditions, the heating rate and the atmosphere. Three distinct stages were observed in Figure 7 for the thermal degradation of well-dried wood fiber under a nitrogen atmosphere. At the beginning, a small step degradation occurred below 100 °C, followed by a second step that occurred at 220–315 °C, and the last one occurred at 315–400 °C. These weight loss steps can be related to the volatilization of the wood extractives and moisture evaporation, the decomposition of the hemicelluloses, and the decomposition of cellulose, respectively [23,41,42]. The other main component of wood fiber, lignin, which composes about 20–30 wt.% of softwoods and hardwoods [43], is the most stable component of thermal decomposition. No specific peak can be recognized for the individual decomposition of the lignin component under the inert atmosphere, since it has a gradual and slow decomposition process throughout the whole temperature range [42]. However, in the case of oxidative degradation, a small peak that occurred at 400–500 °C was observed in Figure 8, which could be related to the degradation of lignin, because almost no cellulose normally remains undegraded above 400 °C [42]. Such a great difference among the decomposition behaviors of the three main components of wood fiber, hemicellulose, cellulose and lignin appears to be related to their inherent chemical structures. An amorphous structure and high content of branches makes hemicellulose degrade easily into carbon-based volatiles at low temperature, whereas the long branchless polymer chains of anhydroglucose account for the higher thermal stability of cellulose. The wide temperature range for the degradation of lignin stems from the presence of various aromatic rings, with different branches covering a wide range of activities for chemical bonds [42].

The thermal degradation of the PP occurred in the range of 300–500 °C, while its oxidative degradation occurred between 250 and 450 °C, as shown in Figure 7 and Figure 8. Figure 7 illustrates that the thermal degradation of the wood–PP biocomposite happened at different stages. Aside from the moisture evaporation below 100 °C, the mass loss was initiated by the degradation of the hemicellulose and cellulose components of the wood fiber, followed by the degradation of the PP. A clear indication is that in the WPC systems, the degradations of the two components can be easily detected individually, owing to the noticeable differences in their thermal stabilities. At a heating rate of 5 °C/min, the *T_5%_* is around 228 °C for wood fiber and 332 °C for PP, whereas it is about 260 °C and 271 °C for WPCs with 40 wt.% and 60 wt.% of wood, respectively. Figure 7a shows that the thermal degradation curves of WPC for the most part are located between the degradation curves of two components, wood fiber and PP, as expected. An almost similar trend was observed for other heating rates (10 °C/min, 20 °C/min, and 40 °C/min) under an inert atmosphere. The degradation temperature peak (*T_peak_*) of each component in the respective zones is tabulated in Table 4. A slight rightward shift in the degradation peak temperature of cellulose and PP is observed in the case of WPCs. This means that when wood fiber was incorporated into the PP matrix, the thermal degradation of each component was slightly delayed. Wood fiber particles are impregnated and covered by the thermoplastic phase, and the considerably higher decomposition temperature of PP, accompanied by the inherently low thermal conductivity of polymers, can delay the degradation of the wood components [24,44]. The slight increase in the *T_peak_* of PP can probably be explained by two mechanisms: the heat sink effect of the residual ash [45] and the thermal insulating effect of the foam-like structure of PP [46]. The first explanation lies in the fact that the residual ash remaining from the degradation of the cellulose and hemicellulose of wood fiber may absorb the heat, and thereby delaying the degradation of the PP component. Secondly, the gas products generated from decomposition of the wood component, predominantly CO_2_ and CO, may be trapped within the PP matrix and form gas cells embedded in the polymer. The resultant foam-like structure inhibits the heat transfer process and suppresses the degradation of PP.

With the exposure of the PP-based WPC to the air atmosphere, the oxidative degradation of all the components commences sooner, as compared to the nitrogen atmosphere. It is interesting to observe in Figure 8 that the *T_5%_* and *T_peak_* of wood and PP^v^W00 become close to each other in the presence of oxygen. The results showed that decreasing the heating rate tends to draw the TGA curves of the two components much closer together, i.e., at the heating rate of 5 °C/min (Figure 8a) the difference between the *T_5%_* of wood fiber and PP is less than 30 °C (~228 °C for wood fiber and 255 °C for PP). The narrow temperature interval between the oxidative degradations of wood fiber and PP is attributed to the partial co-occurrence of their degradations in a wide temperature range. The DTG curves shown in Figure 8 confirm the overlapping tails of the degradation peaks corresponding to cellulose and PP. This means that a separation of the degradation processes of wood and PP could not be achieved simply via the original degradation curves under air.

**Figure 9:**
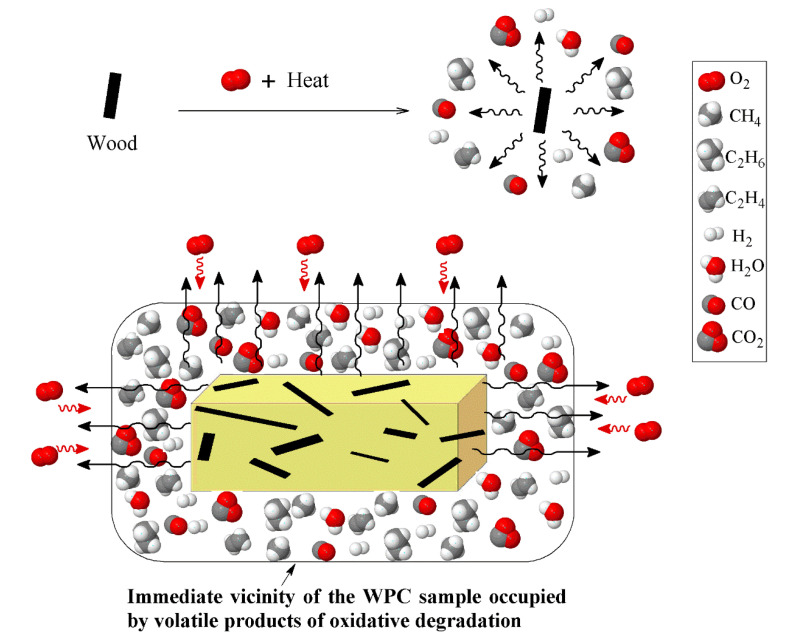
Schematic of reduction in access oxygen due to the presence of volatile products of wood oxidative degradation.

Another important observation in the evolution of the oxidative degradation of WPCs, considering the influence of heating rate, is the location of the degradation curve. Interestingly, at the heating rate of 5 °C/min, the degradation profile of WPC did not fall between the PP and wood fiber curves. However, by increasing the heating rate, the curves were gradually moved towards the area in between the two components. The occurrence of the oxidative degradation of WPC at higher temperatures causes confusion, regarding how the higher thermal stability of WPC than PP and sawdust, in such a wide temperature range (gray zone), could be interpreted. The key point that needs to be taken into account here is the need for oxygen in oxidative degradation. At the low heating rate, 5 °C/min, the oxidative degradation of PP was initiated at ca. 30 °C after that of wood fiber. The volatile compounds that evolved in the oxidative degradation of the wood fiber reduced the access of oxygen species to the polymer chains, as shown in Figure 9. With the lack of access to oxygen, PP requires a higher temperature to decompose than is required for normal oxidative degradation.

Moreover, the antioxidant properties of lignin can improve the stability of PP against photo- and thermo-oxidation [47]. Canetti et al. [48] reported that an increase in the T_5%_ of the oxidative degradation of PP in the presence of lignin can be related to the protective surface formed by the interactions between the PP and the charring lignin, which is able to reduce the oxygen diffusion towards the PP bulk. Thus, it is plausible that the observation of the WPC mass loss curve outside the range of its components could be a result of the limited access to oxygen. By increasing the heating rate, the temperature interval between the oxidative degradations of PP and wood fiber widens, providing a sufficient gap for a significant drop in the concentration of volatiles evolving from the wood fiber, which provides adequate oxygen for PP degradation.

**Figure 10:**
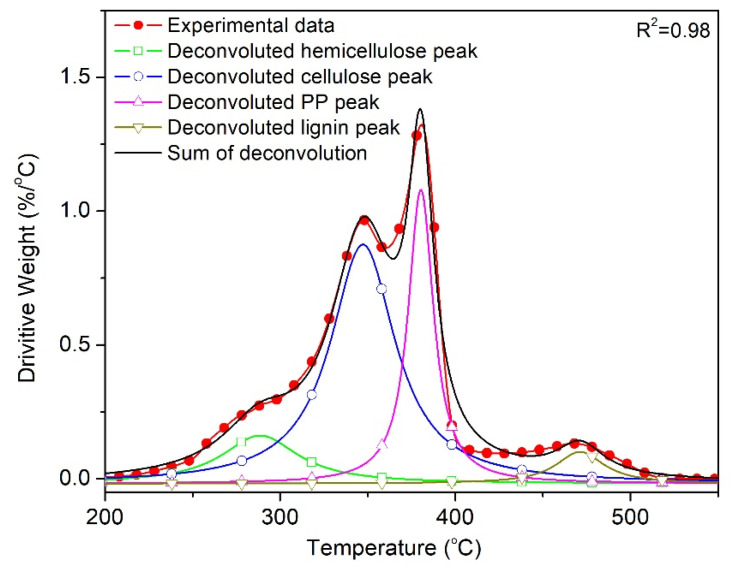
Deconvolution of the DTG curve corresponding to the oxidative degradation of PP^V^W40 at *β* = 10 °C/min.

To conduct a more precise analysis of the effect of wood on the degradation of PP, the DTG curves were deconvoluted to separate the overlapped cellulose and PP peak, and describe the evolution of the decomposition of PP individually. Figure 10 presents the deconvoluted DTG curve corresponding to the thermo-oxidative degradation of PP^V^W40 at a heating rate of 10 °C/min, according to the Lorentzian area deconvolution method. This tool gave an *R^2^* value of 0.98 (inserted in the figure) for the deconvolution, confirming that the employed tool is appropriate and provides an excellent fit between the experimental and calculated results. The evolution of the degree of conversion at any temperature was calculated by dividing the partial area under the peak and the baseline at any temperature by the total area under the peak.

**Figure 11:**
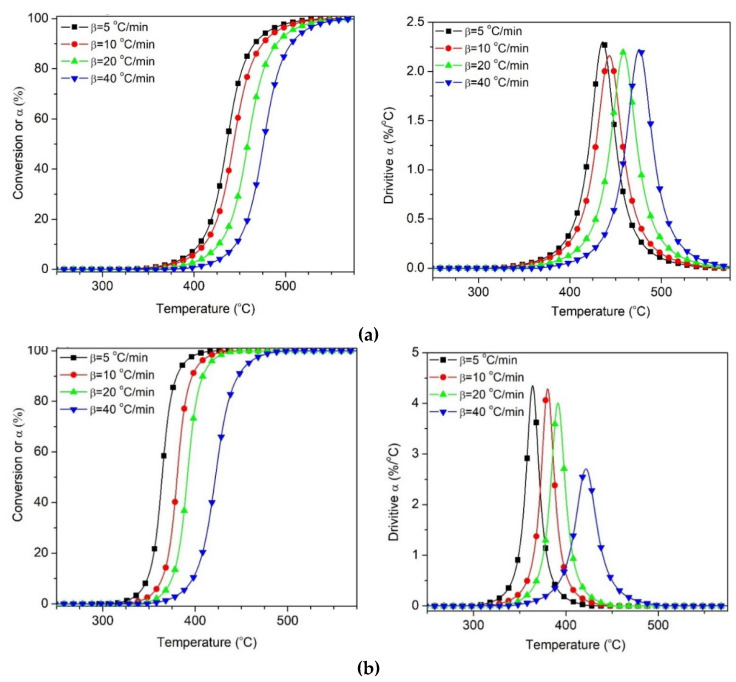
Separated PP degradation obtained from deconvolution of PP^V^W40 under different atmosphere (**a**) Nitrogen, (**b**) Air.

The convoluted PP degradation peak, and the calculated degrees of conversion evolution for the PP^v^W40 samples under inert and reactive atmospheres, are presented in Figure 11. Similar results were obtained for the PP^v^W60 sample, as shown in Supporting Information Figure S4.

**Figure 12:**
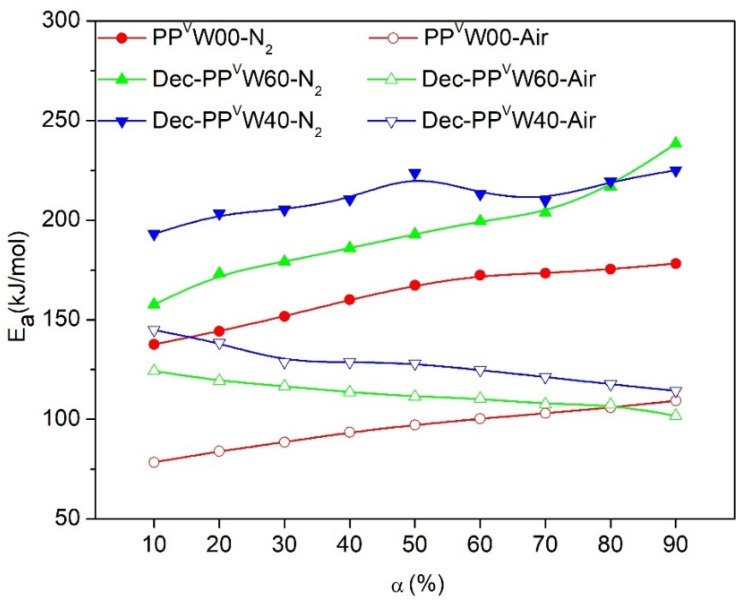
Variation of *E_a_* for PP^v^W00 and individual degradation of PP obtained from deconvolution of PP^V^W40 and PP^V^W60 according to OFW model, Dec- stands for deconvoluted

The values of the deconvoluted figures were employed to determine the *E_a_* as proposed by the iso-conversional models. For instance, the plots of the FWO method, which was employed for the calculation of the *E_a_* values of PP deconvoluted from WPCs, are illustrated in Figure 12. Under an inert environment, the shapes of the *E_a_* evolution curves for PP degradation deconvoluted from WPCs were similar to those of the PP sample without wood (PP^v^W00). However, the upward shift of *E_a_* throughout the whole range of conversions demonstrated that the thermal degradation of individual PP is influenced by the introduction of wood. This behavior may be associated with the high thermal absorbency of the residual chars, and also the high thermal insulating effect of the foam-like structure of PP (PP matrix with some voids filled by ash). Under the air, however, the *E_a_* evolution curve of PP deconvoluted from WPCs showed an irregular decreasing trend with the conversion. A significant increase in *E_a_* values was observed at the initial steps of the oxidative degradation of PP, at conversion values below ca. 40%, where there was an overlap in the oxidative degradations of PP and cellulose. This may be related to the presence of a high volume of volatiles from the degradation of wood, which reduced the oxygen diffusion. The values of *E_a_* during the later stages indicated that the decomposition of PP became almost independent of the wood content, with almost the same *E_a_* value as that of pure PP. The presence of O_2_ enhanced the decomposition of the char from the decomposition of wood in inert conditions, leaving behind no residue. Thus, the effect of wood on the oxidative degradation of PP at high conversions could be considered negligible.

Despite the efforts presented here, the study of the overlapping areas in the degradation of WPCs is still in its preliminary stages, and future research efforts are required to gain an accurate understanding of the degradation phenomenon of each component in the presence of the other one. However, the results of this study provide useful insights into the thermal and oxidative degradation of the polyolefins, through either the recycling of plastics or the manufacturing bio-based composites.

## 4. Conclusions

The simulated degradation behaviors of recycled PP and wood–PP biocomposites have been analyzed by TGA. Three iso-conversional methods, KAS, OFW and Friedman, and two conversion-independent methods, Kissinger and Augis, were employed to calculate *E_a_*, *T_5%_* and *T_peak_* reduction. The results revealed that the PP underwent substantial degradation after reprocessing, confirmed by a remarkable drop in the *τ_∞_* value accompanied by an increase in MFR. A progressive reduction was observed for the *E_a_* value with successive reprocessing cycles, and with a whole range of conversions.

In the WPC systems, two probable mechanisms (heat sink effect of the residual ash and thermal insulating effect of the foam-like structure of PP) were proposed to cause a slight increase in the *T_peak_* of PP during thermal degradation. An interesting observation concerning the oxidation of WPCs was that the TGA profile did not fall between PP and wood curves at low heating rates. The reduction in oxygen accessed by the polymer chains, due to the evolved volatile compounds from wood, was the most probable reason for this. The overlapping of the tails of the degradation peaks corresponding to cellulose and PP was observed in the degradation of WPCs. The Lorentzian area deconvolution method employed to separate them indicated that under N_2_, the *E_a_* evolution curves for PP shifted upward in the whole range of conversions, due to the thermal absorbency of the residual chars. Under the air, a significant increase in *E_a_* values was observed at the initial steps of degradation, which was associated with the high volume of degradation volatiles generated by the wood, and which reduced the oxygen diffusion. At high conversions, the effect of wood became negligible as there were no residuals remaining from the decomposition of wood.

## Figures and Tables

**Table 1:** The composition of the Wood–plastic composites (WPC) samples.

Sample Code	PP Resin (wt.%)	Wood (wt.%)
PP^v*^	100	0
PP^×1^ (PP^v^W00)	100	0
PP^×3^	100	0
PP^×5^	100	0
PP^v^W40	60	40
PP^v^W60	40	60
Wood	0	100

^v^: Virgin; ^×n^: n-time processed.

**Table 2:** Kinetic models used to calculate the activation energy (**E_a_**).

Kinetic Model	Equation	$E_{a}\;\mathrm{Calculation}$
Kissinger–Akahira–Sunose (KAS)	$\ln\left(\frac{\beta }{T^{2}}\right)=\ln\left(\frac{AR}{E_{a}}\right)-\frac{E_{a}}{RT}$	Slope of $\ln\left(\frac{\beta }{T^{2}}\right)$ vs. $\frac{1}{RT}$ at constant conversion (α)
Kissinger	$\ln\left(\frac{\beta }{T_{P}{}^{2}}\right)=\ln\left(\frac{AR}{E_{a}}\right)-\frac{E_{a}}{RT_{P}}$	Slope of $\ln\left(\frac{\beta }{T_{P}{}^{2}}\right)$ vs. $\frac{1}{RT_{P}}$
Ozawa–Flynn–Wall (OFW)	$\log\beta =-0.4567\frac{E_{a}}{RT}+cte$	Slope of logβ vs. $\frac{0.4567}{RT}$ at constant α
Friedman	$\ln\left(\beta \frac{d\alpha }{dT}\right)=\ln A+\ln f\left(\alpha \right)-\frac{E_{a}}{RT}$	Slope of $\ln\left(\beta \frac{d\alpha }{dT}\right)$ vs. $\frac{1}{RT}$ at constant α
Augis	$\ln\left(\frac{\beta }{T_{P}-T_{0}}\right)=\ln A-\frac{E_{a}}{RT_{P}}$	Slope of $\ln\left(\frac{\beta }{T_{P}-T_{0}}\right)$ vs. $\frac{1}{RT_{P}}$

β: Heating rate; $T_{P}$: Peak temperature of DTG; $T_{0}$ or $T_{onset}$: Temperature with 5% of weight loss.

**Table 3:** Characteristic degradation temperatures, *T_5%_* and *T_peak_*, of PP and its recyclates.

Sample	Heating Rate (°C/*min*)	N_2_ Atmosphere		Air Atmosphere	
		*T_5%_* (°C)	*T_peak_* (°C)	*T_5%_* (°C)	*T_peak_* (°C)
PP^v^	5	339.1	431.2	263.6	365.2
	10	351.9	440.6	274.1	398.1
	20	359.1	463.5	299.4	402.0
	40	416.4	487.9	324.6	442.8
PP^×1^	5	332.3	430.5	254.6	331.7
	10	344.5	440.1	262.3	351.1
	20	358.7	460.0	294.6	394.2
	40	386.2	479.0	318.9	430.1
PP^×3^	5	330.7	429.0	246.8	324.3
	10	337.6	438.7	260.2	348.0
	20	354.7	456.2	288.1	385.0
	40	381.3	476.8	312.4	427.4
PP^×5^	5	327.4	423.5	238.5	308.7
	10	334.7	436.7	257.9	338.6
	20	353.1	454.8	285.6	378.9
	40	371.6	474.3	309.7	426.2 s

**Table 4:** *T_peak_* of PP, wood and PP-based WPCs.

Sample	Heating Rate (°C/min)	Atmosphere	*T_peak_* (°C)			
			Hemicellulose	Cellulose	Lignin	PP
**Wood**	**5 °C/min**	N_2_	290.3	335.96	NA	-
		Air	267.9	308.5	442.2	-
**PP^V^W00**		N_2_	-	-	-	430.5
		Air	-	-	-	331.7
**PP^V^W40**		N_2_	291.2	342.8	NA	435.7
		Air	288.3	337.4	456.4	364.0
**PP^V^W60**		N_2_	291.4	344.5	NA	439.6
		Air	290.1	336.7	453.9	364.5
**Wood**	**10 °C/min**	N_2_	293.3	347.5	NA	-
		Air	280.0	321.0	464.1	-
**PP^V^W00**		N_2_	-	-	-	440.1
		Air	-	-	-	351.1
**PP^V^W40**		N_2_	295.1	349.6	NA	441.9
		Air	288.9	347.2	471.1	380.3
**PP^V^W60**		N_2_	296.3	351.8	NA	449.6
		Air	288.7	342.4	468.4	374.4
**Wood**	**20 °C/min**	N_2_	311.5	359.5	NA	-
		Air	292.5	335.7	482.2	-
**PP^V^W00**		N_2_	-	-	-	460.0
		Air	-	-	-	394.2
**PP^V^W40**		N_2_	317.6	368.0	NA	464.8
		Air	308.4	358.2	496.1	393.4
**PP^V^W60**		N_2_	319.5	370.5	NA	468.6
		Air	309.3	356.5	475.4	394.1
**Wood**	**40 °C/min**	N_2_	322.4	372.1	NA	-
		Air	305.2	350.2	501.1	-
**PP^V^W00**		N_2_	-	-	-	479.0
		Air	-	-	-	430.1
**PP^V^W40**		N_2_	331.0	380.1	NA	485.2
		Air	311.0	370.4	536.4	425.7
**PP^V^W60**		N_2_	334.8	384.0	NA	486.1
		Air	310.8	369.3	535.7	422.5

## Supplementary Materials

The following are available online at https://www.mdpi.com/2073-4360/12/8/1627/s1; Figure S1. FTIR spectrum of PP and its recyclates; Figure S2. Processing of polypropylene (a) Evolution of torque and (b) MFR of PP as a function of the number of processing cycles; Figure S3. TG and DTG curves for the effect of reprocessing cycles on the decomposition of PP at various heating rates: (a) 10 °C/min under N_2_, (b) 40 °C/min under N_2_, (c) 10 °C/min under the air, (d) 40 °C/min under O_2_; Figure S4. Separated PP degradation obtained from deconvolution of PPVW60 under different atmospheres: (a) Nitrogen, (b) Air.
