# Supplementary material for: Degradation Behavior of Polypropylene during Reprocessing and Its Biocomposites: Thermal and Oxidative Degradation Kinetics
Source: Polymers (Basel). 2020 Jul 22;12(8):1627. doi: 10.3390/polym12081627 (PMC7464851; doi:10.3390/polym12081627)
Supplement: Supplementary file 1 [file polymers-12-01627-s001.pdf]

Supporting Information

Degradation behavior of polypropylene and its biocomposites during reprocessing: thermal and oxidative degradation kinetics

Elnaz Esmizadeh, Costas Tzoganakis, and Tizazu H. Mekonnen*

Department of Chemical Engineering, Institute of Polymer Research, University of Waterloo, Waterloo, ON, Canada

*Corresponding Author(s) – E-mail: tmekonnen@uwaterloo.ca

Content

S1. Chemical variation of PP after the recycling process

S2. The effect of processing cycle number on mixing torque and melt flow index of PP

S3. The effect of reprocessing cycles on the degradation of PP at heating rates of 10 oC/min and 40 °C/min

S4. The convoluted PP degradation peak and the calculated degree of conversion evolution for PP^vW60 sample under the inert and reactive atmosphere

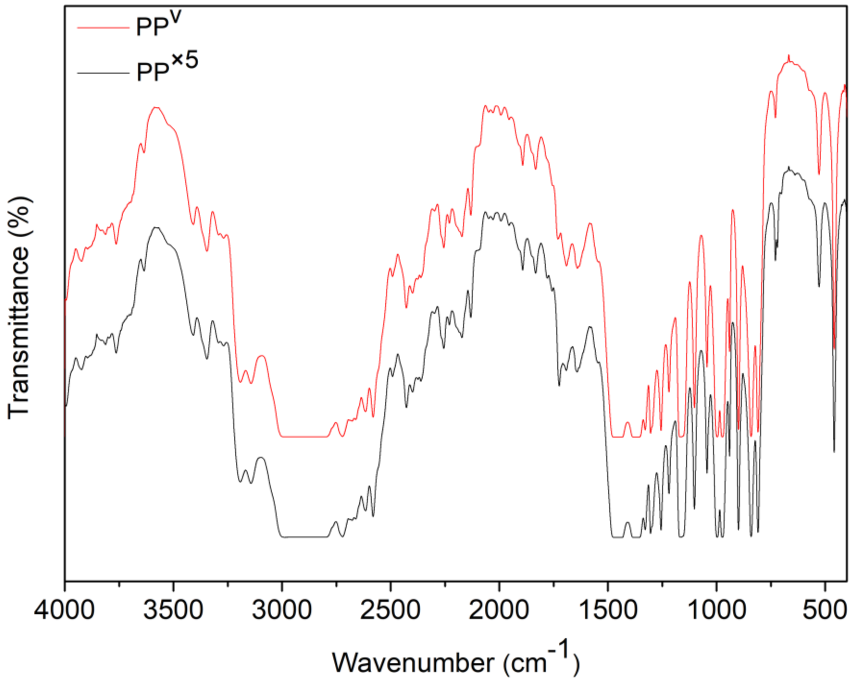

Figure S1. FTIR spectrum of PP and its recyclates

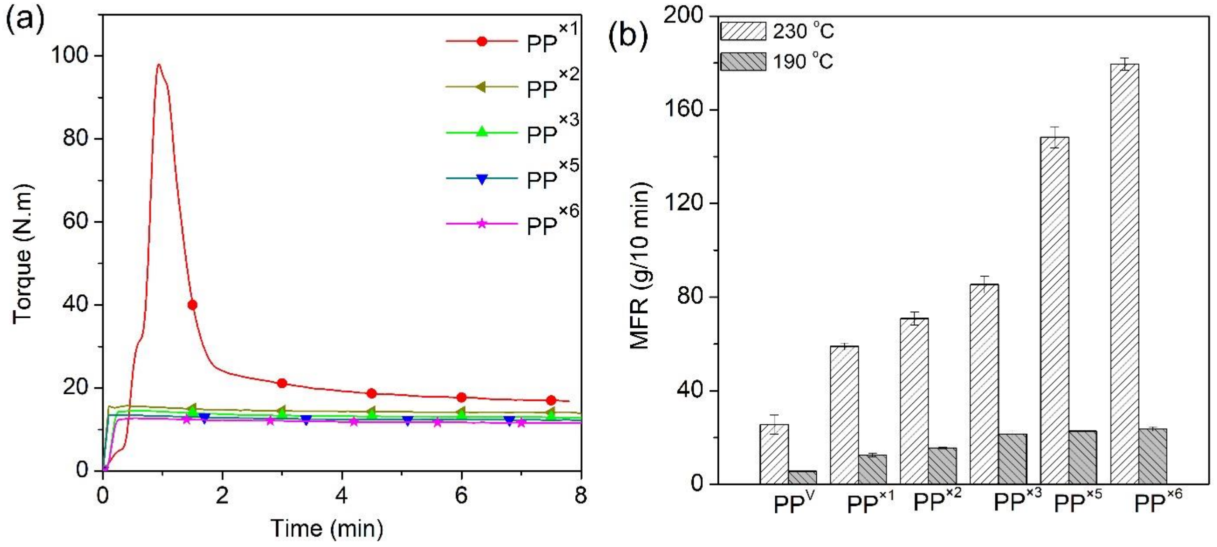

Figure S2. Processing of polypropylene (a) Evolution of torque and (b) MFR of PP as a function of the number of processing cycles

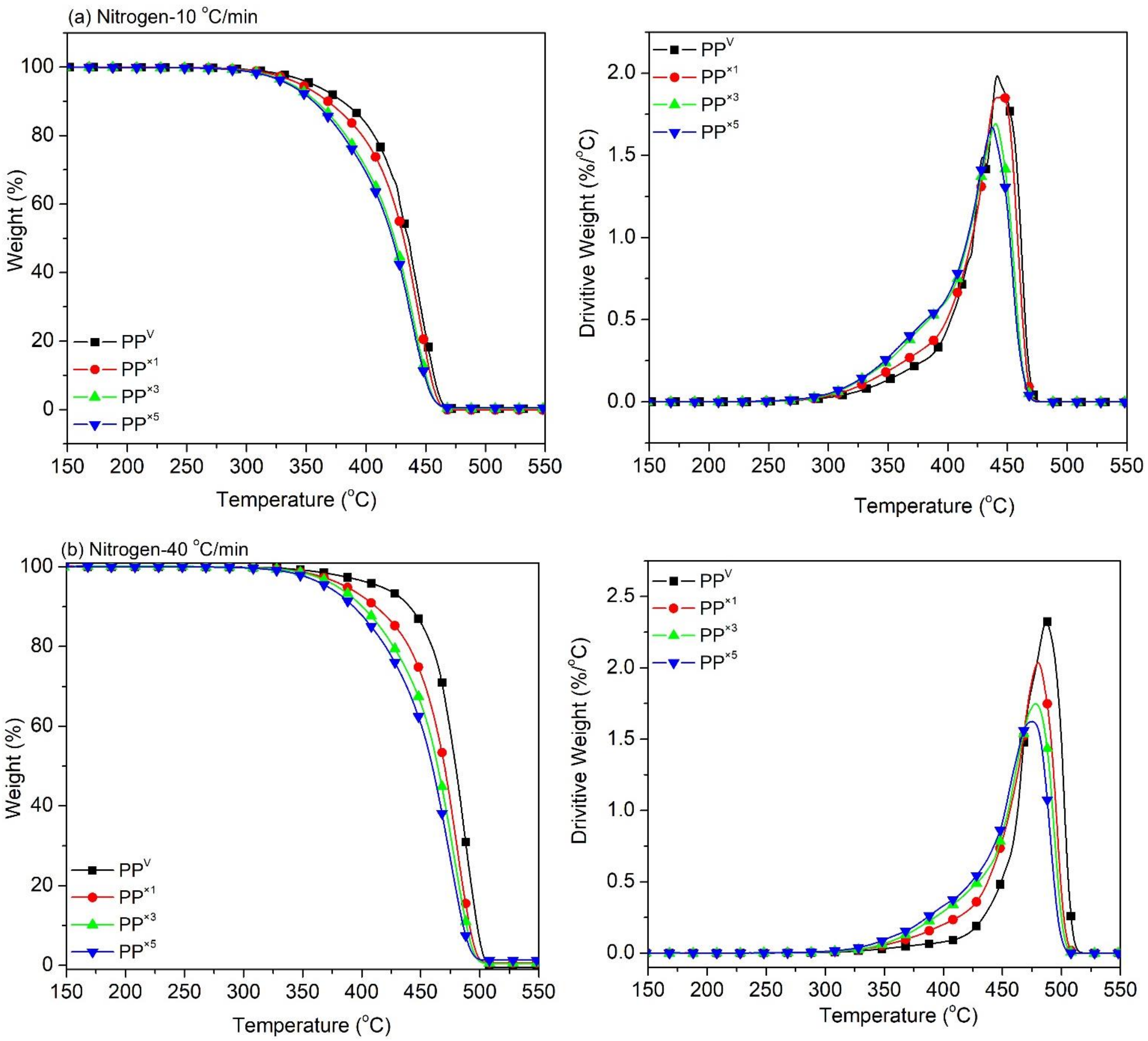

(a) Nitrogen-10 °C/min
Weight (%)
Temperature (°C)
Drivitive Weight (%/°C)
PP^{V}
$PP^{\times1}$
$PP^{\times3}$
$PP^{\times5}$
(b) Nitrogen-40 °C/min
Weight (%)
Temperature (°C)
Drivitive Weight (%/°C)
PP^{V}
$PP^{\times1}$
$PP^{\times3}$
$PP^{\times5}$

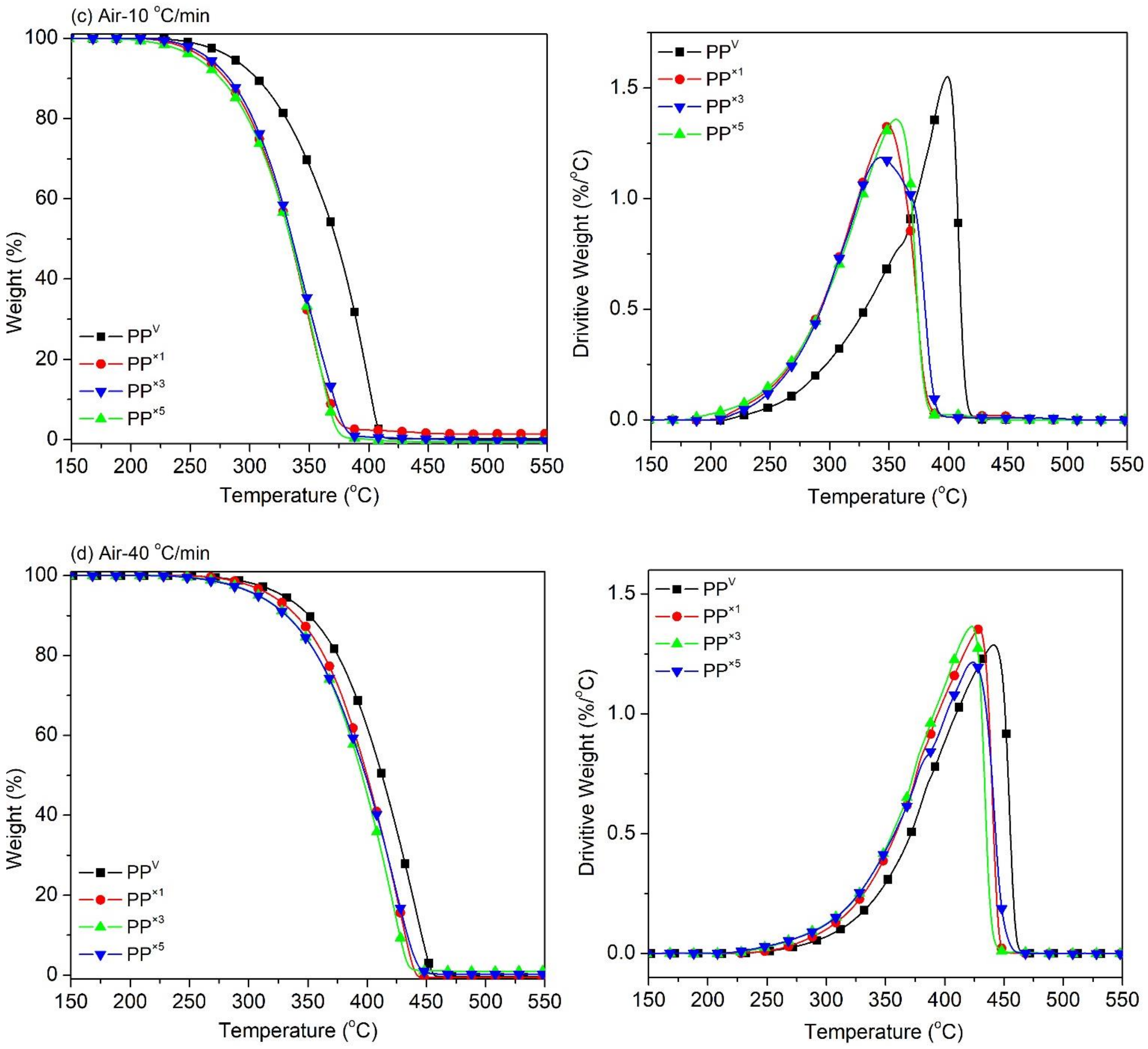

Figure S3. TG and DTG curves for the effect of reprocessing cycles on the decomposition of PP at various heating rates: (a)10 ºC/min under N_2, (b) 40 ºC/min under N_2, (c)10 ºC/min under the air, (d) 40 ºC/min under O_2

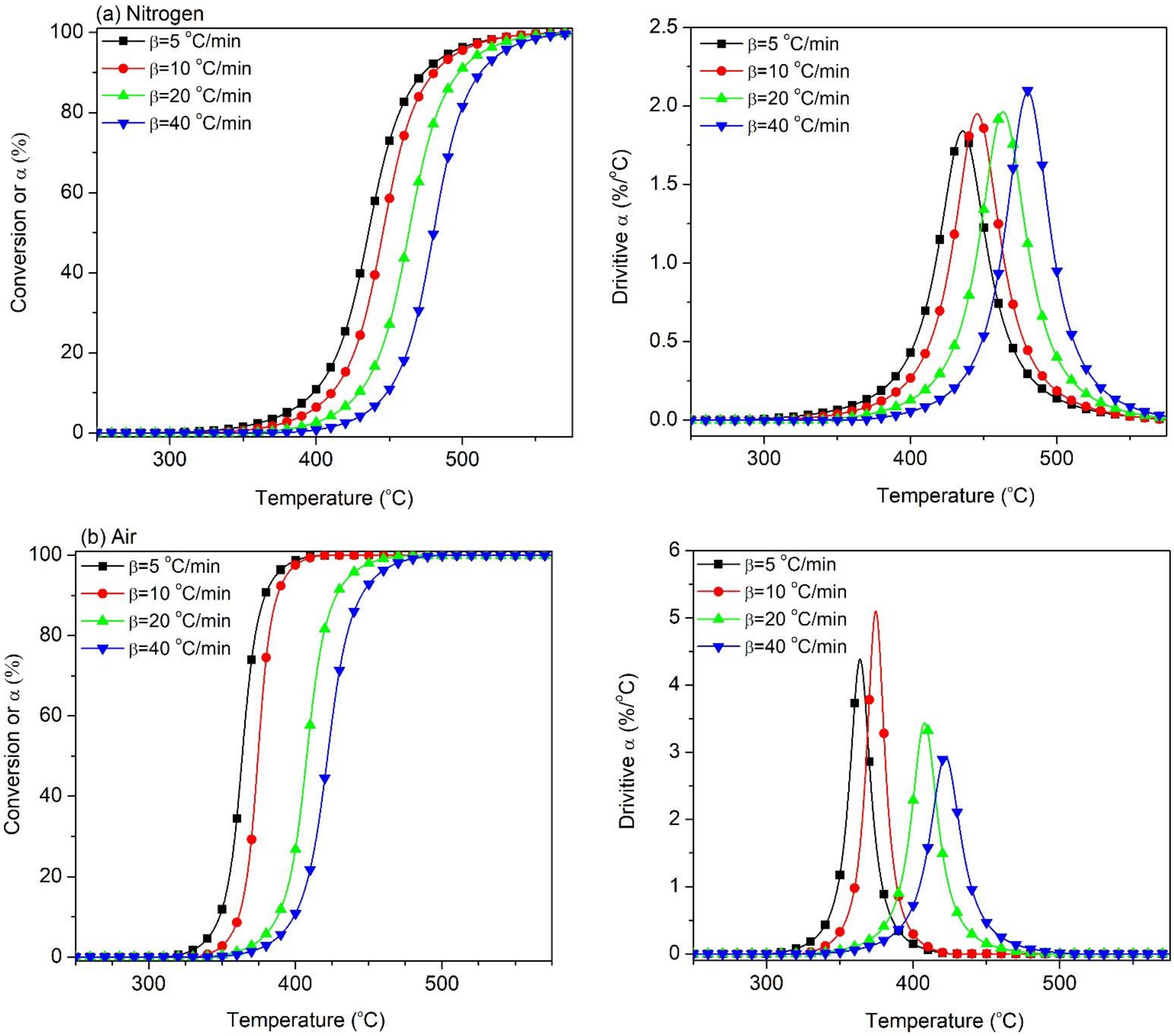

Figure S4. Separated PP degradation obtained from deconvolution of $PP^{V}W60$ under different atmospheres: (a) Nitrogen, (b) Air
